# Association of circadian rhythms with brain disorder incidents: a prospective cohort study of 72242 participants

**DOI:** 10.1038/s41398-022-02278-1

**Published:** 2022-12-14

**Authors:** Si-Jia Chen, Yue-Ting Deng, Yu-Zhu Li, Ya-Ru Zhang, Wei Zhang, Shi-Dong Chen, Bang-Sheng Wu, Liu Yang, Qiang Dong, Jianfeng Feng, Wei Cheng, Jin-Tai Yu

**Affiliations:** 1 Department of Neurology and National Center for Neurological Disorders, Huashan Hospital, State Key Laboratory of Medical Neurobiology and MOE Frontiers Center for Brain Science, Shanghai Medical College, Fudan University, Shanghai, China; 2 Institute of Science and Technology for Brain-inspired Intelligence, Fudan University, Shanghai, China

**Keywords:** Depression, Psychiatric disorders

## Abstract

Circadian rhythm disruption (CRD) is a shared characteristic of various brain disorders, such as Alzheimer’s disease (AD), Parkinson’s disease (PD), and major depression disorder (MDD). Disruption of circadian rhythm might be a risk factor for brain disorder incidents. From 7-day accelerometry data of 72,242 participants in UK Biobank, we derived a circadian relative amplitude variable, which to some extent reflected the degree of circadian rhythm disruption. Records of brain disorder incidents were obtained from a wide range of health outcomes across self-report, primary care, hospital inpatient data, and death data. Using multivariate Cox proportional hazard ratio regression, we created two models adjusting for different covariates. Then, linear correlations between relative amplitude and several brain morphometric measures were examined in participants with brain MRI data. After a median follow-up of around 6.1 years, 72,242 participants were included in the current study (female 54.9%; mean age 62.1 years). Individuals with reduced relative amplitude had increasing risk of all-cause dementia (Hazard ratio 1.23 [95% CI 1.15 to 1.31]), PD (1.33 [1.25 to 1.41]), stroke (1.13 [1.06 to 1.22]), MDD (1.18 [1.13 to 1.23]), and anxiety disorder (1.14 [1.09 to 1.20]) in fully adjusted models. Additionally, significant correlations were found between several cortical regions and white matter tracts and relative amplitude that have been linked to dementia and psychiatric disorders. We confirm CRD to be a risk factor for various brain disorders. Interventions for regulating circadian rhythm may have clinical relevance to reducing the risk of various brain disorders.

## Introduction

Endogenous circadian rhythms are internal oscillations found in the human body and brain, which dominate almost all vital physiology and metabolism with a period of approximately a day [1]. These rhythms, predominantly regulated by molecular clockworks within the hypothalamic suprachiasmatic nucleus (SCN), help to maintain the homeostasis of the whole brain functions via synaptic and endocrine factors [2]. Circadian rhythm disruption (CRD), which means dysfunction in 24-h circadian rhythms, is a common occurrence in aging adults. It has been reportedly linked to various brain disorders, particularly neurodegenerative and psychiatric disorders [3, 4]. While CRD may interact with neurodegeneration in Alzheimer’s disease (AD) or Parkinson’s disease (PD) and form a deleterious cycle [5], it may also be a major component of many mood, anxiety, and psychotic disorders [6]. Stroke, as a common vascular brain disorder, is less considered to be directly connected with CRD, but some sleep disorders, such as obstructive sleep apnea syndrome (OSAS), have been widely thought to be a risk factor for stroke [7]. In recent years, various therapies that improve circadian rhythm have proven effective in improving these brain disorders, including non-pharmacological treatments (light-therapy) [8, 9] and pharmacological treatments (agomelatine, melatonin) [10–13]. These facts indicate that CRD may play an important role in the pathophysiology of these disorders.

Although quite a few studies have explored the associations between circadian rhythm and several common brain disorders, most of them are cross-sectional, enroll relatively small or highly selected samples, adopt subjective sleep measures, or adjust for a few confounders [14–18]. Until now, there are still few articles investigating the longitudinal association of CRD with these disorders, whereas CRD could be a preclinical sign for adjuvant diagnosis or a potential target for prevention [6].

Recently, the collection of accelerometer data from over 90,000 participants in UK Biobank means an unprecedented opportunity for a prospective, large-scale observational study to determine the potential influence of CRD on various brain disorders. The detailed baseline information on demographic, lifestyle, and genetics also makes it possible to control for a wide range of potential confounders within multivariable models.

Here, we perform a prospective cohort study of 72242 UK Biobank participants to investigate the association of circadian rhythms with common brain disorders. Given the prevalence, clinical relevance, and biological correlation, a broad spectrum of brain disorders is examined in our analyses, including all-cause dementia, AD, PD, stroke, major depression disorder (MDD), and anxiety disorders.

## Methods

### Participants

Over half a million participants aged 37–73 were recruited into the UK Biobank from 2006 to 2010. Through 22 assessment centers, information on a participant’s demographic, lifestyle, health, and physical assessments and questionnaires were collected at the baseline [19]. The current study includes only a subset of 103,683 participants that provided accelerometer data using a wrist-worm accelerometer between 2013 and 2015 (Start time of wear: Field ID 90011; End time of wear: Field ID 90011) [20]. UK biobank was approved by the North West Multi-Center Research Ethics Committee (ref 11/NW/03820) and all participants provided written informed consent.

### Measurement of circadian rhythm

Between 2013 and 2015, 103,683 participants accepted the invitation to wear a wrist-worn AX3 triaxial accelerometer for 7 days and provided objective physical activity data. The data were then pre-processed by the UKB accelerometer expert working group before releasing to UK Biobank [20]. The details are available on the UK Biobank Data Showcase website. We excluded individuals with insufficient wear time (<72 h of wear time or no wear data in each 1 h period of the 24 h cycle), or poor device calibration identified by UK Biobank.

As a part of circadian rhythms, the rest-activity pattern indicates circadian rhythmicity, reflecting the timing and regularity of physical activity and sleep [21]. Relative amplitude is a common and convenient non-parametric measure of the amplitude of rest-activity rhythm calculated from the most active continuous 10 h (M10, “day”) and the least active continuous 5 h (L5, “night”) in an average 24 h [22, 23]. The computing formula is as follows:

$${\rm{Relative}}\;{\rm{amplitude}} = \frac{{(M10 - L5)}}{{(M10 + L5)}}$$

As it suggests, relative amplitude ranges from 0 to 1. Higher values reflect a clearer distinction between activity levels during the most and least active periods of the day, on the contrary, lower values indicate disrupted circadian rhythms. We derived relative amplitude by processing raw accelerometer data in UK Biobank (field ID 90004) [22].

### Covariates

At the baseline assessment, participants provided data on demographic characteristics including age, sex, educational attainment, Townsend deprivation indices, ethnic origin, lifestyle, and genotype. Townsend social deprivation scores were derived based on the postcode of residence, with negative scores reflecting relatively greater affluence [24]. Educational attainment was first converted into years of education according to the self-reported educational qualification from a touchscreen questionnaire [25] and then recoded into an ordinal scale from 0 to 10 (e.g., 0: 0 years; 10: >24 years) [26]. More details can be found in previous studies. Smoking status and alcohol consumption status (never, previous, or current) were derived from a touchscreen lifestyle questionnaire. Wear season is the time that accelerometry starts (spring for March to May, summer for June to August, autumn for September to November, and winter for December to February; UK Meteorological Office definitions). According to a modified version of the International Physical Activity Questionnaire (IPAQ), physical activity was collected and transformed into a continuous variable (MET-minutes/week) [27]. Body-mass index (BMI) was calculated from measurements of height (m) and weight (kg) using weight divided by height^2^. *ApoE-ε4* (apolipoprotein E, type epsilon4 allele) genotype after quality control and imputation by UK Biobank was dichotomized to the carrier (one or two alleles) versus non-carrier. Further information on the genotyping process is available on the website [28]. These covariates were selected based on prior potential associations with circadian disruption, outcome-related variables, or both [29–34].

### Measurement of brain disorders

The outcomes were defined as the first occurrence of seven common brain disorders including dementia, AD, PD, stroke, MDD, and anxiety disorder. All outcome data were obtained from the first occurrence of health outcomes defined by 3-character ICD-codes (UK biobank category 1712), which are generated from a wide range of health outcomes across self-report, primary care, hospital inpatient data, and death data. Self-report of health conditions is from the Biobank assessment clinics in four instances. Primary care and hospital inpatient data are real-world, administrative data captured during the delivery of care. And death-related data is received from the National Health Service (NHS). The details of the data processing and linkage are available on the UK Biobank website [35]. Using the International Classification of Diseases, 10th version (ICD-10), all-cause dementia was defined as F00 (dementia in Alzheimer’s disease), F01 (vascular dementia), F02 (dementia in other diseases) or F03 (unspecified dementia), and G30 (Alzheimer’s disease), among which AD was F00 or G30. Others were, respectively, PD (G20), stroke (I60-I69), MDD (F32, F33), and anxiety disorders (F40, F41).

The start of follow-up was the final date of accelerometer wear for each participant. Participants with any of these seven brain disorders before that date were excluded from the analysis. The end of the follow-up was the first occurrence of these brain disorders or the end of the study (31 December 2021).

### Brain MRI data

Since 2014, brain MRI data from over 40,000 UK Biobank participants have been collected on the same 3T Siemens Skyra scanner with a 32-channel head coil according to a freely available protocol [36, 37]. T1-weighted images were processed and analyzed by the UK Biobank imaging team using the FSL diffusion tensor fitting program (FMRIB) Software library version 6.0 [38, 39], and made as distinct imaging-derived phenotypes (IDPs), including atlas regions’ surface area, volume and mean thickness, as well as subcortical volume and global measures of fractional anisotropy (FA). The full details of the imaging processing and quality control pipeline are available in an open-access article [38]. We included participants who have both MRI and actigraphy data, and excluded those with any major brain disorder before the end of the study. Finally, the surface area, volume, and mean thickness of 68 cortical regions, the volume of 36 subcortical regions, and the FA of 15 white matter tracts from 11,901 participants were included to investigate the associations between the relative amplitude and detailed aspects of brain macrostructure and microstructure.

### Statistical analyses

Characteristics of participants were compared by groups of the relative amplitude using *χ*^2^ tests for categorical variables, analysis of variance for normally distributed continuous variables, and Kruskal–Wallis tests for continuous variables with skewed distributions. Hazard ratios (HRs) for associations between the relative amplitude predictor and each of the brain disorders were examined using multivariable-adjusted Cox proportion hazard regression models. The Bonferroni correction was employed for multiple comparisons.

First, we included the relative amplitude as a continuous variable in the regression model to find the overall trend. The relative amplitude scores were first inverted by multiplying by –1 and standardized with the HR expressing the influence of lower relative amplitude per 1-SD (standard deviation) increase. Second, to emphasize the influence of low relative amplitude, we divided relative amplitude into low, medium, and high categories with mean ± SD as demarcation.

Basic analyses (Model 1) were adjusted for age, sex, Townsend deprivation index, ethnicity (white or non-white), and wear season. Model 2 additionally adjusted for educational attainment, smoking (never, former, and current), alcohol (never, former, and current), physical activity, BMI, and *ApoE-ε4* allele.

Furthermore, to validate the robustness of the models, two sensitivity analyses were performed. One additionally adjusted for sleep disorders (First occurrence ICD-10: G47) before the final date of accelerometer wear, and another removed the participants with self-reported experience of shift work at the baseline assessment centers (Field ID: 3426). We also conducted prespecified subgroup analyses stratified by sex, age, and *ApoE-ε4*. According to age, the participants were divided into two groups: <65 years old and ≥65 years old as the demarcation of adults and elderly. According to *ApoE-ε4*, the participants were divided into two groups: *ApoE-ε4* carrier and *ApoE-ε4* non-carrier.

Assuming that circadian rhythm is possibly linked to pathological changes in certain brain regions, the associations between regional MRI measures (cortical area, thickness and volume, subcortical volume, and white matter tract-specific FA) and continuous relative amplitude were examined by multiple linear regression after adjusting for the same covariates as above. The results were corrected for multiple comparisons using the false discovery rate (FDR).

R 3.6.1 (using the packages ‘survival’, and ‘survminer’) were used to perform the analyses. A *p*-value below 0.05 was considered statistically significant.

## Results

### Population characteristics

**Fig. 1:**
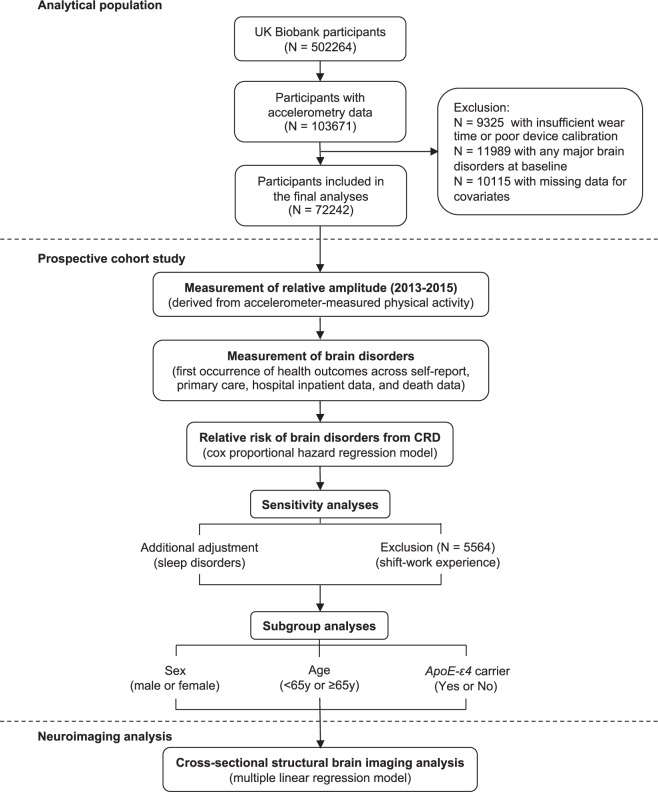
The analytical pipeline of the study. We totally included 72242 participants from UK Biobank with accelerometer data. First, we examined the associations between relative amplitude and brain disorders using the Cox proportion hazard regression model. Second, we examined the associations between relative amplitude and brain MRI measures by multiple linear regression.

After excluding participants who had the brain disorders we study and who had missing values of the covariates at baseline, 72,242 individuals were included in this prospective study (Fig. 1). The cohort characteristics by relative amplitude categories are presented (Table 1). We computed descriptive statistics—mean (SD) for continuous variables and percentage for categorical variables. Overall, individuals with lower relative amplitude were older, more likely to be female, from deprived socioeconomic status, and current smokers. They were also more likely to have higher BMI.

**Table 1:** Characteristic of the cohort.

Characteristics	Overall (*n* = 72,242)	Relative amplitude			*p*-value^a^
		Low (*n* = 9194)	Median (*n* = 55,061)	High (*n* = 7987)	
Age (years), mean (SD)	62.1 (7.8)	64.8 (7.3)	62.1 (7.7)	59.0 (7.8)	<0.001
Female sex, *n* (%)	39,670 (54.9)	4029 (43.8)	31,311 (56.9)	4330 (54.2)	<0.001
ApoE4 allele carrier, *n* (%)	20,382 (28.2)	2527 (27.5)	15,517 (28.2)	2338 (29.3)	0.076
Education, mean (SD)^b^	5.5 (2.4)	5.4 (2.4)	5.5 (2.4)	5.6 (2.4)	<0.001
Townsend, mean (SD)	−1.9 (2.7)	−1.4 (3.0)	−2.0 (2.6)	−2.1 (2.6)	<0.001
Ethnic_White, *n* (%)	72,240 (100.0)	9194 (100.0)	55,060 (100.0)	7986 (100.0)	0.204
Smoking status, *n* (%)					<0.001
Never	41,968 (58.1)	4675 (50.8)	32,275 (58.6)	5018 (62.8)	
Former smoker	25,766 (35.7)	3607 (39.2)	19,524 (35.5)	2635 (33.0)	
Current smoker	4508 (6.2)	912 (9.9)	3262 (5.9)	334 (4.2)	
Alcohol consumption status, *n* (%)					<0.001
Never	1692 (2.3)	284 (3.1)	1245 (2.3)	163 (2.0)	
Former drinker	1705 (2.4)	344 (3.7)	1194 (2.2)	167 (2.1)	
Current drinker	68,845 (95.3)	8566 (93.2)	52,622 (95.6)	7657 (95.9)	
Wear_season, *n* (%)					<0.001
Spring	25,483 (35.3)	3203 (34.8)	19,391 (35.2)	2889 (36.2)	
Summer	19,000 (26.3)	2324 (25.3)	14,518 (26.4)	2158 (27.0)	
Autumn	21,280 (29.5)	2723 (29.6)	16,255 (29.5)	2302 (28.8)	
Winter	6479 (9.0)	944 (10.3)	4897 (8.9)	638 (8.0)	
BMI (kg/m^2^), mean (SD)	26.6 (4.4)	29.0 (5.4)	26.4 (4.2)	25.1 (3.5)	<0.001
Events, *n* (%)					
All-cause dementia	262 (0.4)	85 (0.9)	163 (0.3)	14 (0.2)	<0.001
Alzheimer’s disease	115 (0.2)	25 (0.3)	81 (0.1)	9 (0.1)	0.011*
Parkinson’s disease	190 (0.3)	70 (0.8)	114 (0.2)	6 (0.1)	<0.001
Stroke	481 (0.7)	111 (1.2)	341 (0.6)	29 (0.4)	<0.001
Depression	1102 (1.5)	219 (2.4)	785 (1.4)	98 (1.2)	<0.001
Anxiety	1161 (1.6)	209 (2.3)	846 (1.5)	106 (1.3)	<0.001

^a^Calculated using *χ*^2^ tests for categorical variables, analysis of variance for normally distributed continuous variables, and Kruskal–Wallis tests for continuous variables with skewed distributions.

^b^Calculated as weight in kilometers divided by square of height in meters.

*p-value not significant after Bonferroni correction (*α* = 0.05/16).

**Fig. 2:**
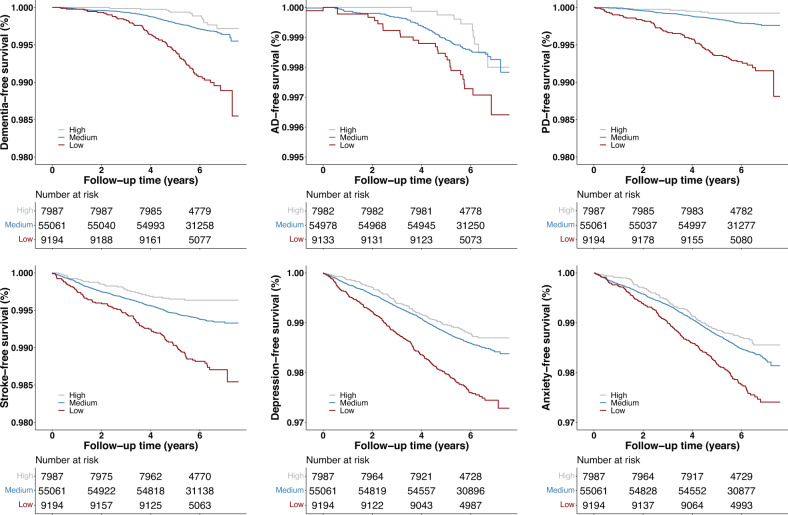
Kaplan–Meier curves for incidence of different brain disorders against time to the occurrence. The low, medium, and high relative amplitude categories were derived from dividing continuous relative amplitude scores with mean ± SD as demarcation.

The median follow-up time for any brain disorders was around 6.1 years with SD 0.6–0.8 years. During the follow-up, 262 individuals were diagnosed with incident dementia, 115 with AD, 190 with PD, 481 with stroke, 1102 with MDD, and 1161 with anxiety disorders (Table 1). Additionally, for each of the brain disorders, Kaplan–Meier curves for each relative amplitude category showed participants in low relative amplitude category had the highest incidence (Fig. 2).

### Relative amplitude and risk of brain disorder incidents

In terms of continuous relative amplitude scores, there was a significant trend towards a higher risk of all-cause dementia (HR 1.22 [95% CI 1.14 to 1.29], *p* < 0.001), PD (HR 1.28 [95% CI 1.22 to 1.35], *p* < 0.001), stroke (HR 1.14 [95% CI 1.07 to 1.22], *p* < 0.001), MDD (HR 1.23 [95% CI 1.18 to 1.27], *p* < 0.001) and anxiety disorder (HR 1.16 [95% CI 1.11 to 1.21], *p* < 0.001) in Model 1 (Table 2). The results with Model 2 were similar to those in Model 1 (Table 2). However, the significant association with AD was only observed in Model 2 (HR 1.16 [95% CI 1.01 to 1.33], *p* < 0.05), which became no longer significant after Bonferroni correction (*α* = 0.05/6).

**Table 2:** Adjusted HRs for the association between continuous relative amplitude and brain disorder incidents.

		Model 1		Model 2	
	Events	HR (95% CI)	*p*-value	HR (95% CI)	*p*-value
All-cause dementia	262	1.22 (1.14–1.29)	<0.001	1.23 (1.15–1.31)	<0.001
Alzheimer’s disease	115	1.12 (0.98–1.28)	0.102	1.16 (1.01–1.33)	0.036*
Parkinson’s disease	190	1.28 (1.22–1.35)	<0.001	1.33 (1.25–1.41)	<0.001
Stroke	481	1.14 (1.07–1.22)	<0.001	1.13 (1.06–1.22)	<0.001
Depression	1102	1.23 (1.18–1.27)	<0.001	1.18 (1.13–1.23)	<0.001
Anxiety	1161	1.16 (1.11–1.21)	<0.001	1.14 (1.09–1.20)	<0.001

Model 1 was adjusted by age, sex, Townsend deprivation index, ethnicity, and wear season. Model 2 was additionally adjusted by educational attainment, smoking status, alcohol consumption status, physical activity, BMI, and *ApoE-ε4* allele.

*p-value not significant after Bonferroni correction (*α* = 0.05/6).

**Fig. 3:**
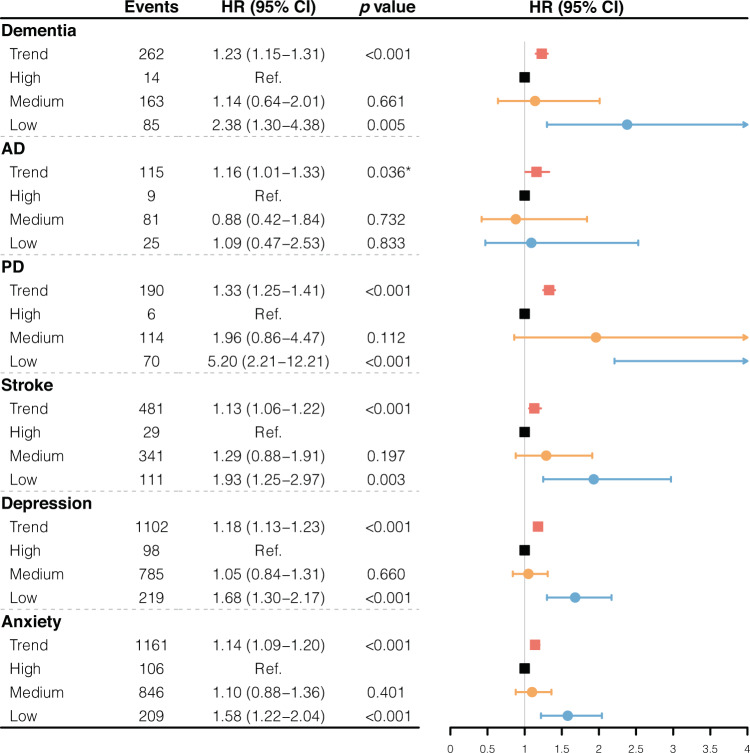
Forest plot of HRs for brain disorder incidents by high, medium, and low relative amplitude. Analyses were adjusted by age, sex, Townsend deprivation index, ethnicity, wear season, educational attainment, smoking status, alcohol consumption status, physical activity, BMI, and *ApoE-ε4* (Model 2).

Compared with individuals in high relative amplitude category, those in low relative amplitude category had a significantly higher risk of all-cause dementia (HR 2.25 [95% CI 1.27 to 3.98], *p* = 0.006), PD (HR 4.91 [95% CI 2.12 to 11.38], *p* < 0.001), stroke (HR 1.96 [95% CI 1.29 to 2.97], *p* = 0.001), MDD (HR 2.10 [95% CI 1.64 to 2.67], *p* < 0.001) and anxiety disorder (HR 1.65 [95% CI 1.30 to 2.10], *p* < 0.001) in Model 1 (Figure S1). And the results remained robust and significant in Model 2 (Fig. 3). However, compared to medium relative amplitude, high amplitude did not reduce the risk significantly despite relatively lower estimated hazard ratios. Further sensitivity analyses additionally adjusting for sleep disorders did not alter the findings, but those excluding participants with shift-work experience were no longer significant in all-cause dementia and stroke after Bonferroni correction (Table S1).

### Relative amplitude and brain disorders risk in subgroup analyses

To investigate whether some covariates played an important role in the associations between relative amplitude and risk of major brain disorders, we performed a series of subgroup analyses. First, subgroup analysis by sex showed that lower relative amplitude was significantly associated with increased stroke risk only in females. And it seemed that relative amplitude was more strongly associated with PD risk in males. Second, subgroup analysis by age showed that low relative amplitude was significantly associated with all-cause dementia and stroke only in the elderly and it was more strongly associated with MDD and anxiety disorder in the adult group. Third, we performed subgroup analysis by *ApoE-ε4* carrier status for all-cause dementia and AD. The outcomes were consistent in two groups. (Tables S2–4).

### Associations between relative amplitude and brain structure

**Fig. 4:**
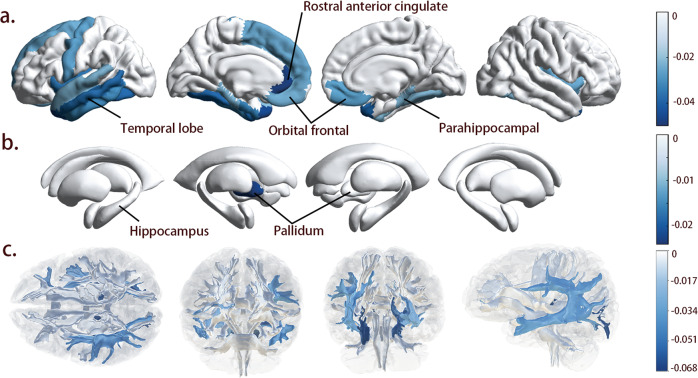
Associations between relative amplitude and regional MRI measures. Analyses were adjusted by age, sex, Townsend deprivation index, ethnicity, and wear season. Initial continuous relative amplitude was inverted by multiplying −1. R-value represents the correlation coefficient of the linear regression. Here, we only show the regions with FDR-corrected *p*-value < 0.05. **a**, **b** Cortical thickness and subcortical volume. **c** White matter tract-specific FA (from left to right: superior, anterior, posterior, and left lateral views).

To further investigate the potential pathway, we investigate the associations between relative amplitude and brain structure. In model 1, FDR-corrected significant associations with cortex emerged where lower relative amplitude with lower thickness was mainly in the limbic lobe (left cingulate gyrus and right parahippocampal gyrus), left temporal lobe, left fusiform lobe, insular lobe, and frontal lobe (bilateral medial orbital frontal cortex and superior frontal cortex) (Fig. 4 and Table S5). However, in model 2, the associations with parahippocampal gyrus, insular lobe, and medial orbital frontal are no longer significant. And significant associations between subcortical volume and relative amplitude were also shown (Table S5). We also conducted association analyses between cortical area and volume and relative amplitude, but no statistically significant correlations were found. Lower relative amplitude was also significantly associated with reduced FA of association fibers (inferior longitudinal fasciculus, superior longitudinal fasciculus, and inferior frontal-occipital fasciculus), and internal capsule (posterior thalamic radiations and right anterior thalamic radiations) in both models (Fig. 4). And the association with cingulate gyrus part of cingulum, anterior thalamic radiation, and superior thalamic radiation is significant only in model 2 (Table S6).

## Discussion

In this study, we found that lower relative amplitude, which may reflect disturbance of circadian rhythm, was a risk factor for a broad spectrum of brain disorders independent of age, sex, educational attainment, Townsend deprivation index, ethnicity, wear-season, smoking status, alcohol consumption status, physical activity, BMI and *ApoE-ε4*. These brain disorders cover neurogenerative disorders (all-cause dementia, and PD), vascular disorders (stroke), and psychiatric disorders (MDD and anxiety disorder). In addition, we discovered brain structural differences in cortical thickness, subcortical volume, and white matter tract-specific FA in regions previously found to be altered in dementia and psychiatric disease.

Although many studies have found individuals with dementia always have disturbed circadian rhythms, like rest-activity rhythm [16, 40], and stronger rhythms indicate less severe dementia [41], few articles have a prospective design to study how CRD influences the occurrence of dementia. Simultaneously, the reduced amplitude of rest-activity is also found in PD patients, who sometimes exhibit symptoms like daytime sleepiness, insomnia, and restless legs syndrome [17], but the effect of CRD on incidence is not addressed. Recently, using the parametric cosine model, two articles, respectively, reported low amplitude as a risk factor for AD and PD both based on prospective cohorts of community-dwelling old people (HR: 1.39 and 1.77) [14, 42]. Our findings are similar using a non-parametric method and a much larger cohort, although we cannot obtain any significant results in AD after Bonferroni correction across the analyses. In the current study, the two age-related neurodegenerative diseases are first put together to compare the effect of CRD. It seems that PD is exactly more strongly affected by CRD than AD (HR: 1.16 and 1.33). In recent years, many studies focusing on circadian rhythm and neurodegenerative disorders have thrown light on similar mechanisms. When the circadian rhythm is disrupted, the physiological functions, such as promoting clearance of misfolded protein, maintaining the brain’s redox homeostasis, and regulating inflammatory activation, are all impaired to varying degrees that initiate or exacerbate neurodegeneration [6, 43, 44]. Convincedly, evidence of animal experiments and analyses of postmortem brain tissues and in vivo cerebrospinal fluid both show altered expression rhythm of a series of well-known clock genes in neurodegeneration [5, 43, 45]. However, the clear pathophysiological pathway is still ambiguous and requires more experiments to find out.

In recent years, increasing stroke incidence has been reported in individuals with various sleep disorders, such as obstructive sleep apnea, sleep-related movement disorders, and insomnia, which contribute to sleep fragmentation, increased nocturnal arousals, or daytime sleepiness [46–48]. However, to our knowledge, it is rarely discussed that circadian rhythm amplitude could be associated with the risk of stroke onset independent of sleep disorders, and our work first exhibits a significant 13% higher risk of incident stroke. In our view, one main pathway is via sympathetic hyperactivity and hypothalamic-pituitary-adrenal axis activation. CRD of sleep can disrupt their normal rhythm and eventually lead to hypertension which is a common risk factor for stroke [49, 50]. And neuroinflammation may mediate this pathway based on the discovery of microglia activation in rodents in hypertension [51].

As for psychiatric disorders, many cross-sectional studies show a low amplitude in current depression and anxiety [15, 16, 52]. We first provide a large-scale prospective cohort study to determine the associations of circadian rhythms with MDD and anxiety at the same time (HR: 1.18 and 1.14). And the estimated HRs of MDD are higher than those of anxiety disorder in any sensitivity or subgroup analyses. Similar biological alterations as neurodegenerative disorders and stroke, including clock gene expression, inflammation cytokine, and sympathetic nervous system are also reported [53, 54], suggesting similar pathways from CRD to various brain disorders. Another hypothetical mechanism is that disrupted sleep-wake rhythm in adolescents affects the synaptic pruning and maturation of neural circuits which could lead to the susceptibility to psychiatric diseases later in life [6]. Although our design is not sufficient to be interpreted from this perspective due to the limitation of the cohort, we believe further research will make more progress.

Our models are still robust after adjustment for sleep disorders which as a form of brain disorders, obviously disrupt our rest-activity rhythm and were reported to be associated with the occurrence of common brain disorders [46, 55, 56]. It indicates that the effect cannot completely be accounted for by individuals with pre-existing sleep disorders. Another sensitivity analysis excluded participants with shift-work experience, which widely prove to be associated with various brain disorders, particularly stroke and mood disorders [57, 58]. After that the associations are no longer significant in all-cause dementia and stroke after Bonferroni correction, it indicates shift-work experience is a noteworthy risk factor for the two disorders. Subgroup analyses stratified by sex shows a stronger association with incident PD in male. It suggests CRD is a more important intervening factor for men. And CRD in the elderly seems more strongly associated with all-cause dementia and stroke, and in the adults with MDD and anxiety disorder. It can be explained that age is an important factor affecting the morbidity of these disorders [59, 60]. There may be an interaction between the effect of CRD and age on the pathogenesis of the incidents as existing evidence indicates diverse biological pathways from CRD to brain disorders at different life stage [6].

To our knowledge, no previous study has investigated the associations of relative amplitude with brain structure. Surprisingly, we find brain macrostructural and microstructural changes that could affect our mental processes. Particularly, the medial orbital cortex involves in the formation of the medial prefrontal network (MPFC) to regulate our affection [61]. The limbic system including the cingulate gyrus and parahippocampal gyrus, coordinates the evoking of emotion, memories, and behavior [62–65]. However, related subcortical nuclei, such as the hippocampus and amygdala are not significantly associated with lower relative amplitude in the current study. In addition, white matter tracts with lower FA that is thought to reflect a lower degree of neuronal organization [66], have been reported to be associated with dementia and psychiatric disorders, and our analyses show that they are also linked to relative amplitude. Limbic and cortico-cortical tracts exist within the framework of the neurocircuitry of mood disorders and anxiety [67, 68]. The integrity of the thalamic connectivity, including anterior and posterior thalamic radiations is also found damaged in dementia and psychiatric disorders [69, 70]. Because of the cross-sectional design, it is challenging to determine the causal relationship between relative amplitude and brain structural changes. However, some potential mechanisms, such as accumulation of misfolded protein, dysregulation of redox homeostasis, and inflammatory activation could all contribute to the brain structural changes [6]. Above all, our findings suggest potential disease-related brain structural changes alone with CRD long before the occurrence of many brain disorders. And due to an unclear timeline and a small number of cases, we fail to conduct mediation analyses. Further prospective neuroimaging evidence may help make the pathway clear.

However, the cohort of UK Biobank is characterized by partial UK residents thought to be older, richer, and healthier, so the risk estimates can be different in a distinct population considering rest-activity rhythm and prevalence of diseases varies with age and areas. Although we take account of a wide range of potential confounders, some unmeasured or residual confounding remains a possibility. Due to relatively short follow-up duration and low incidence of AD, the results of dementia subtype analyses may have insufficient power. Therefore, future research with longer follow-up of the UK Biobank cohort is needed. In this study, we make relative amplitude represent the status of the whole follow-up duration, while within-participant changes may have a profound impact on the final results. Future collection of accelerometry data from the same people is supposed to complement and extend the current study. In the meanwhile, the relative amplitude cannot completely reflect our rest-activity pattern, let alone circadian rhythm. However, the convenience makes it more available in practice. Given that this is an observational study, it cannot figure out a definite causal relationship. Future research about the underlying biological mechanism is needed to support the results.

### Conclusions

In conclusion, our findings further confirm that CRD is a risk factor for developing common neurodegenerative and psychiatric disorders, including all-cause dementia, AD, PD, stroke, MDD, and anxiety disorder. Although the mechanism underlying these associations remains to be elucidated, we prove brain structural changes related to brain disorders in people with low relative amplitude long before the occurrence of brain disorders by analyzing brain MRI data. Future researchers should pay more attention to circadian rhythm considering it is a potential target for preventing various brain disorders. And the relative amplitude of rest-activity pattern, as a simple and valid measure of circadian rhythmicity, is promising to be applied in clinical practice.

## Supplementary information

Sumpplemental Material

## Data Availability

The dataset supporting the conclusions of this article is available in the UKB repository (https://www.ukbiobank.ac.uk/). All code used for data preparation and analysis are available upon request.
