# Supplementary material for: Association of circadian rhythms with brain disorder incidents: a prospective cohort study of 72242 participants
Source: Transl Psychiatry. 2022 Dec 14;12:514. doi: 10.1038/s41398-022-02278-1 (PMC9751105; doi:10.1038/s41398-022-02278-1)
Supplement: Supplementary file 1 — Sumpplemental Material [file 41398_2022_2278_MOESM1_ESM.docx]

**Supplementary Materials**

**Association of circadian rhythms with brain disorder incidents: a prospective study of 72242 participants**

[Figure S1. Forest plot of HRs for brain disorder incidents by high, medium and low relative amplitude. 2](#_Toc117176748)

[Table S1. Adjusted HRs for brain disorder incidents by high, medium and low relative amplitude in two sensitivity analyses. 3](#_Toc117176749)

[Table S2. Adjusted HRs for brain disorder incidents by high, medium and low amplitude stratified by sex. 4](#_Toc117176750)

[Table S3. Adjusted HRs for brain disorder incidents by high, medium and low amplitude stratified by age. 5](#_Toc117176751)

[Table S4. Adjusted HRs for brain disorder incidents by high, medium and low amplitude stratified by *ApoE-ε4*. 5](#_Toc117176752)

[Table S5. Linear correlation coefficients of thickness of 68 cortical regions and 36 subcortical regions with relative amplitude. 6](#_Toc117176753)

[Table S6. Linear correlation coefficients of white matter tract-specific FA with relative amplitude. 9](#_Toc117176754)

# Figure S1. Forest plot of HRs for brain disorder incidents by high, medium and low relative amplitude.


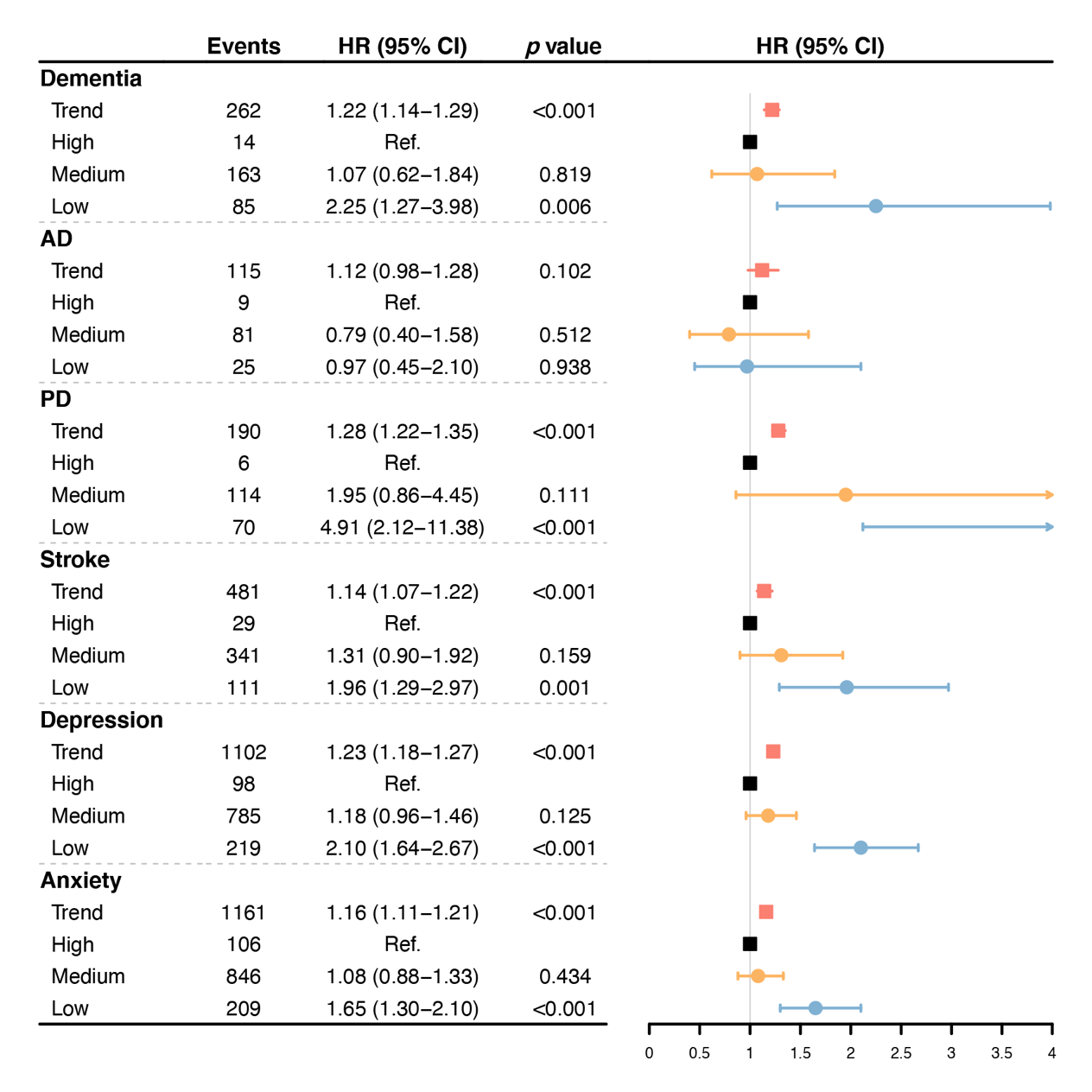


Analyses were adjusted by age, sex, Townsend deprivation index, ethnicity and wear-season (Model 1). **p-value* cannot pass Bonferroni correction (*α* = 0.05/6).

# Table S1. Adjusted HRs for brain disorder incidents by high, medium and low relative amplitude in two sensitivity analyses.

|  | **sleep disorders** | | **shift-work experience** | |
| --- | --- | --- | --- | --- |
| **Relative amplitude** | **HR (95%CI)** | ***p-value*** | **HR (95%CI)** | ***p-value*** |
| **Dementia** |  |  |  |  |
| Trend | 1.23 (1.15-1.31) | **<0.001** | 1.29 (1.06-1.58) | 0.012* |
| High | Ref. |  | Ref. |  |
| Medium | 1.14 (0.64-2.01) | 0.663 | 1.63 (0.50-5.29) | 0.420 |
| Low | 2.38 (1.30-4.37) | **0.005** | 3.03 (0.85-10.83) | 0.088 |
| **Alzheimer's disease** |  |  |  |  |
| Trend | 1.16 (1.01-1.33) | 0.032* | 1.24 (0.87-1.77) | 0.226 |
| Low | Ref. |  | Ref. |  |
| Medium | 0.88 (0.42-1.84) | 0.739 | 2.33 (0.31-17.59) | 0.411 |
| High | 1.11 (0.48-2.57) | 0.811 | 1.64 (0.16-16.56) | 0.674 |
| **Parkinson's disease** |  |  |  |  |
| Trend | 1.33 (1.25-1.41) | **<0.001** | 1.48 (1.30-1.70) | **<0.001** |
| High | Ref. |  | Ref. |  |
| Medium | 1.96 (0.86-4.47) | 0.112 | 1.49 (0.45-4.88) | 0.512 |
| Low | 5.20 (2.22-12.22) | **<0.001** | 5.20 (1.50-17.96) | 0.009* |
| **Stroke** |  |  |  |  |
| Trend | 1.13 (1.06-1.22) | **0.001** | 1.18 (1.03-1.35) | 0.017* |
| High | Ref. |  | Ref. |  |
| Medium | 1.29 (0.88-1.91) | 0.197 | 1.13 (0.65-1.99) | 0.663 |
| Low | 1.93 (1.25-2.97) | **0.003** | 1.97 (1.03-3.75) | 0.040* |
| **Depression** |  |  |  |  |
| Trend | 1.18 (1.13-1.23) | **<0.001** | 1.14 (1.05-1.24) | **0.001** |
| High | Ref. |  | Ref. |  |
| Medium | 1.05 (0.84-1.31) | 0.657 | 1.09 (0.81-1.46) | 0.572 |
| Low | 1.66 (1.28-2.15) | **<0.001** | 1.73 (1.20-2.49) | **0.003** |
| **Anxiety** |  |  |  |  |
| Trend | 1.14 (1.09-1.20) | **<0.001** | 1.13 (1.03-1.23) | **0.008** |
| High | Ref. |  | Ref. |  |
| Medium | 1.10 (0.88-1.36) | 0.403 | 0.93 (0.71-1.22) | 0.605 |
| Low | 1.57 (1.21-2.03) | **0.001** | 1.52 (1.07-2.15) | 0.019* |

Two analyses were respectively performed by correction of sleep disorders and excluding patients with shift-work experience (N = 5564) at baseline. Analyses were adjusted by age, sex, Townsend deprivation index, ethnicity, wear-season, educational attainment, smoking status, alcohol consumption status, physical activity, BMI and *ApoE-ε4*. **p-value* cannot pass Bonferroni correction (*α* = 0.05/6).

# Table S2. Adjusted HRs for brain disorder incidents by high, medium and low amplitude stratified by sex.

|  | **Male** | | **Female** | |
| --- | --- | --- | --- | --- |
| **Relative amplitude** | **HR (95%CI)** | ***p-value*** | **HR (95%CI)** | ***p-value*** |
| **Dementia** |  |  |  |  |
| Trend | 1.29 (1.13-1.48) | **<0.001** | 1.21 (1.12-1.31) | **<0.001** |
| High | Ref. |  | Ref. |  |
| Medium | 0.83 (0.40-1.74) | 0.623 | 1.62 (0.65-4.03) | 0.296 |
| Low | 1.83 (0.81-4.14) | 0.146 | 3.31 (1.29-8.50) | 0.013* |
| **AD** |  |  |  |  |
| Trend | 1.07 (0.81-1.42) | 0.621 | 1.19 (1.03-1.38) | 0.019* |
| Low | Ref. |  | Ref. |  |
| Medium | 0.46 (0.20-1.04) | 0.061 | 3.87 (0.53-28.35) | 0.183 |
| High | 0.60 (0.21-1.69) | 0.330 | 4.67 (0.60-36.52) | 0.142 |
| **PD** |  |  |  |  |
| Trend | 1.61 (1.44-1.80) | **<0.001** | 1.29 (1.19-1.39) | **<0.001** |
| High | Ref. |  | Ref. |  |
| Medium | 1.51 (0.36-6.38) | 0.576 | 2.18 (0.80-5.98) | 0.130 |
| Low | 7.52 (1.69-33.35) | **0.008** | 4.65 (1.64-13.18) | **0.004** |
| **Stroke** |  |  |  |  |
| Trend | 1.09 (0.94-1.26) | 0.272 | 1.15 (1.06-1.24) | **0.001** |
| High | Ref. |  | Ref. |  |
| Medium | 1.63 (0.86-3.10) | 0.137 | 1.10 (0.68-1.80) | 0.692 |
| Low | 1.85 (0.88-3.90) | 0.106 | 1.89 (1.11-3.22) | 0.020* |
| **Depression** |  |  |  |  |
| Trend | 1.22 (1.14-1.30) | **<0.001** | 1.14 (1.07-1.21) | **<0.001** |
| High | Ref. |  | Ref. |  |
| Medium | 1.12 (0.85-1.47) | 0.426 | 0.93 (0.65-1.34) | 0.711 |
| Low | 1.70 (1.22-2.38) | **0.002** | 1.59 (1.06-2.38) | 0.026* |
| **Anxiety** |  |  |  |  |
| Trend | 1.15 (1.07-1.24) | **<0.001** | 1.14 (1.06-1.22) | **<0.001** |
| High | Ref. |  | Ref. |  |
| Medium | 1.08 (0.84-1.40) | 0.545 | 1.13 (0.77-1.67) | 0.530 |
| Low | 1.52 (1.10-2.09) | 0.011* | 1.71 (1.10-2.66) | 0.017* |

Analyses were adjusted by age, Townsend deprivation index, ethnicity, wear-season, educational attainment, smoking status, alcohol consumption status, physical activity, BMI and *ApoE-ε4*. **p-value* cannot pass Bonferroni correction (*α* = 0.05/6).

# Table S3. Adjusted HRs for brain disorder incidents by high, medium and low amplitude stratified by age.

|  | **<65y** | | **≥65y** | |
| --- | --- | --- | --- | --- |
| **Relative amplitude** | **HR (95%CI)** | ***p-value*** | **HR (95%CI)** | ***p-value*** |
| **Dementia** |  |  |  |  |
| Trend | 1.26 (0.93-1.70) | 0.140 | 1.23 (1.15-1.31) | **<0.001** |
| High | Ref. |  | Ref. |  |
| Medium | 1.53 (0.35-6.64) | 0.573 | 1.06 (0.57-1.97) | 0.844 |
| Low | 1.75 (0.28-11.11) | 0.553 | 2.31 (1.21-4.44) | 0.012* |
| **Alzheimer's disease** |  |  |  |  |
| Trend | 1.18 (0.61-2.30) | 0.626 | 1.15 (1.00-1.33) | 0.049* |
| Low | Ref. |  | Ref. |  |
| Medium | NA | NA | 0.75 (0.36-1.58) | 0.450 |
| High | NA | NA | 1.00 (0.43-2.32) | 0.994 |
| **Parkinson's disease** |  |  |  |  |
| Trend | 1.35 (1.08-1.70) | 0.009* | 1.34 (1.26-1.42) | **<0.001** |
| High | Ref. |  | Ref. |  |
| Medium | 1.92 (0.45-8.14) | 0.379 | 1.97 (0.72-5.38) | 0.188 |
| Low | 3.17 (0.63-15.97) | 0.162 | 5.82 (2.08-16.26) | **0.001** |
| **Stroke** |  |  |  |  |
| Trend | 1.07 (0.90-1.28) | 0.462 | 1.15 (1.07-1.24) | **<0.001** |
| High | Ref. |  | Ref. |  |
| Medium | 1.01 (0.57-1.79) | 0.961 | 1.53 (0.89-2.63) | 0.125 |
| Low | 1.56 (0.77-3.17) | 0.217 | 2.27 (1.27-4.05) | **0.005** |
| **Depression** |  |  |  |  |
| Trend | 1.21 (1.13-1.29) | **<0.001** | 1.16 (1.09-1.24) | **<0.001** |
| High | Ref. |  | Ref. |  |
| Medium | 1.17 (0.89-1.54) | 0.267 | 0.81 (0.57-1.16) | 0.257 |
| Low | 2.14 (1.53-2.98) | **<0.001** | 1.14 (0.76-1.70) | 0.535 |
| **Anxiety** |  |  |  |  |
| Trend | 1.20 (1.11-1.29) | **<0.001** | 1.12 (1.04-1.20) | **0.002** |
| High | Ref. |  | Ref. |  |
| Medium | 1.21 (0.91-1.61) | 0.185 | 0.92 (0.67-1.28) | 0.632 |
| Low | 1.89 (1.33-2.70) | **<0.001** | 1.28 (0.88-1.86) | 0.196 |

Analyses were adjusted by sex, Townsend deprivation index, ethnicity, wear-season, educational attainment, smoking status, alcohol consumption status, physical activity, BMI and *ApoE-ε4*. **p-value* cannot pass Bonferroni correction (*α* = 0.05/6).

# Table S4. Adjusted HRs for brain disorder incidents by high, medium and low amplitude stratified by *ApoE-ε4*.

|  | **ApoE4 non-carrier** | | **ApoE4 carrier** | |
| --- | --- | --- | --- | --- |
| **Relative amplitude** | **HR (95%CI)** | ***p-value*** | **HR (95%CI)** | ***p-value*** |
| **Dementia** |  |  |  |  |
| Trend | 1.23 (1.13-1.34) | **<0.001** | 1.21 (1.08-1.37) | **0.001** |
| High | Ref. |  | Ref. |  |
| Medium | 1.40 (0.56-3.48) | 0.471 | 1.07 (0.46-2.49) | 0.872 |
| Low | 3.43 (1.32-8.89) | 0.011* | 1.92 (0.77-4.78) | 0.161 |
| **AD** |  |  |  |  |
| Trend | 1.21 (1.01-1.44) | 0.036* | 1.15 (0.93-1.41) | 0.201 |
| Low | Ref. |  | Ref. |  |
| Medium | 1.44 (0.34-6.07) | 0.622 | 0.86 (0.3-2.44) | 0.771 |
| High | 2.24 (0.47-10.56) | 0.308 | 1.02 (0.31-3.42) | 0.973 |

Analyses were adjusted by age, sex, Townsend deprivation index, ethnicity, wear-season, educational attainment, smoking status, alcohol consumption status, physical activity and BMI. **p-value* cannot pass Bonferroni correction (*α* = 0.05/6).

# Table S5. Linear correlation coefficients of thickness of 68 cortical regions and 36 subcortical regions with relative amplitude.

| **Model 1** | **Left** | | **Right** | |
| --- | --- | --- | --- | --- |
| **Brain regions** | **coefficient** | ***p-value*** | **coefficient** | ***p-value*** |
| **Cortical area** |  |  |  |  |
| rostralanteriorcingulate thickness | -0.047 | **0.000** | -0.014 | 0.241 |
| temporalpole thickness | -0.046 | **0.000** | -0.039 | **0.000** |
| fusiform thickness | -0.038 | **0.000** | -0.022 | **0.044** |
| middletemporal thickness | -0.037 | **0.000** | -0.019 | 0.075 |
| insula thickness | -0.035 | **0.001** | -0.026 | **0.018** |
| entorhinal thickness | -0.033 | **0.002** | -0.016 | 0.147 |
| inferiortemporal thickness | -0.031 | **0.003** | -0.019 | 0.085 |
| precentral thickness | -0.027 | **0.011** | -0.008 | 0.527 |
| superiorfrontal thickness | -0.025 | **0.020** | -0.007 | 0.545 |
| medialorbitofrontal thickness | -0.024 | **0.023** | -0.027 | **0.011** |
| parahippocampal thickness | -0.023 | **0.037** | -0.022 | **0.042** |
| superiortemporal thickness | -0.022 | **0.042** | -0.013 | 0.268 |
| lateralorbitofrontal thickness | -0.020 | 0.075 | -0.015 | 0.180 |
| supramarginal thickness | -0.019 | 0.085 | 0.005 | 0.685 |
| posteriorcingulate thickness | -0.016 | 0.162 | -0.013 | 0.256 |
| caudalanteriorcingulate thickness | -0.014 | 0.239 | 0.009 | 0.445 |
| bankssts thickness | -0.010 | 0.394 | -0.014 | 0.241 |
| precuneus thickness | -0.009 | 0.463 | -0.003 | 0.829 |
| paracentral thickness | -0.009 | 0.473 | -0.004 | 0.749 |
| caudalmiddlefrontal thickness | -0.008 | 0.527 | -0.001 | 0.967 |
| parsopercularis thickness | -0.005 | 0.668 | 0.011 | 0.394 |
| isthmuscingulate thickness | -0.002 | 0.878 | -0.008 | 0.489 |
| parstriangularis thickness | -0.002 | 0.878 | 0.003 | 0.824 |
| transversetemporal thickness | -0.002 | 0.878 | 0.003 | 0.829 |
| parsorbitalis thickness | 0.000 | 0.995 | 0.006 | 0.653 |
| frontalpole thickness | 0.006 | 0.637 | 0.028 | **0.009** |
| inferiorparietal thickness | 0.006 | 0.618 | 0.010 | 0.401 |
| superiorparietal thickness | 0.009 | 0.473 | 0.030 | **0.005** |
| rostralmiddlefrontal thickness | 0.009 | 0.445 | 0.019 | 0.075 |
| postcentral thickness | 0.013 | 0.256 | 0.030 | **0.005** |
| lingual thickness | 0.025 | **0.020** | 0.039 | **0.000** |
| lateraloccipital thickness | 0.026 | **0.013** | 0.039 | **0.000** |
| pericalcarine thickness | 0.038 | **0.000** | 0.057 | **0.000** |
| cuneus thickness | 0.046 | **0.000** | 0.058 | **0.000** |
| **Subcortical area** |  |  |  |  |
| cerebellum Cortex | -0.041 | **0.000** | -0.037 | **0.001** |
| ventralDC | -0.035 | **0.001** | -0.036 | **0.001** |
| pallidum | -0.023 | **0.039** | -0.020 | 0.074 |
| vessel | -0.010 | 0.432 | -0.007 | 0.650 |
| thalamus proper | -0.010 | 0.448 | -0.007 | 0.648 |
| choroid plexus | -0.002 | 0.991 | 0.000 | 1.000 |
| hippocampus | -0.002 | 0.991 | -0.004 | 0.835 |
| accumbens area | 0.012 | 0.332 | 0.013 | 0.283 |
| lateral ventricle | 0.014 | 0.214 | 0.016 | 0.149 |
| amygdala | 0.016 | 0.149 | 0.025 | **0.025** |
| inferior lateral ventricle | 0.020 | 0.074 | 0.024 | **0.027** |
| putamen | 0.021 | 0.055 | 0.020 | 0.073 |
| caudate | 0.024 | **0.026** | 0.029 | **0.007** |
| brain stem | -0.032 | **0.003** |  |  |
| CC Posterior | -0.028 | **0.010** |  |  |
| CC Anterior | -0.025 | **0.024** |  |  |
| CC Mid Posterior | -0.017 | 0.121 |  |  |
| CC Central | -0.015 | 0.181 |  |  |
| fifth ventricle | -0.008 | 0.550 |  |  |
| CC Mid Anterior | -0.001 | 1.000 |  |  |
| forth ventricle | 0.000 | 1.000 |  |  |
| optic chiasm | 0.002 | 0.991 |  |  |
| third ventricle | 0.018 | 0.098 |  |  |

| **Model 2** | **Left** | | **Right** | |
| --- | --- | --- | --- | --- |
| **Brain regions** | **coefficient** | ***p-value*** | **coefficient** | ***p-value*** |
| **Cortical area** |  |  |  |  |
| rostralanteriorcingulate thickness | -0.036 | **0.002** | -0.007 | 0.652 |
| temporalpole thickness | -0.028 | **0.030** | -0.025 | **0.042** |
| fusiform thickness | -0.025 | **0.042** | -0.015 | 0.259 |
| middletemporal thickness | -0.026 | **0.041** | -0.015 | 0.259 |
| insula thickness | -0.021 | 0.096 | -0.016 | 0.256 |
| entorhinal thickness | -0.018 | 0.176 | -0.010 | 0.499 |
| inferiortemporal thickness | -0.021 | 0.096 | -0.014 | 0.311 |
| precentral thickness | -0.028 | **0.027** | -0.008 | 0.636 |
| superiorfrontal thickness | -0.026 | **0.041** | -0.010 | 0.524 |
| medialorbitofrontal thickness | -0.017 | 0.208 | -0.024 | 0.057 |
| parahippocampal thickness | -0.016 | 0.242 | -0.014 | 0.330 |
| superiortemporal thickness | -0.011 | 0.426 | -0.005 | 0.756 |
| lateralorbitofrontal thickness | -0.017 | 0.240 | -0.009 | 0.541 |
| supramarginal thickness | -0.019 | 0.163 | 0.004 | 0.794 |
| posteriorcingulate thickness | -0.010 | 0.499 | -0.009 | 0.541 |
| caudalanteriorcingulate thickness | -0.006 | 0.721 | 0.015 | 0.259 |
| bankssts thickness | -0.005 | 0.756 | -0.013 | 0.336 |
| precuneus thickness | -0.012 | 0.385 | -0.006 | 0.721 |
| paracentral thickness | -0.003 | 0.842 | 0.000 | 0.991 |
| caudalmiddlefrontal thickness | -0.011 | 0.414 | -0.007 | 0.636 |
| parsopercularis thickness | -0.007 | 0.652 | 0.007 | 0.636 |
| isthmuscingulate thickness | 0.001 | 0.957 | -0.007 | 0.651 |
| parstriangularis thickness | -0.008 | 0.600 | -0.004 | 0.778 |
| transversetemporal thickness | -0.006 | 0.721 | -0.002 | 0.882 |
| parsorbitalis thickness | -0.003 | 0.796 | 0.000 | 0.978 |
| frontalpole thickness | -0.005 | 0.756 | 0.014 | 0.330 |
| inferiorparietal thickness | 0.004 | 0.794 | 0.005 | 0.756 |
| superiorparietal thickness | -0.003 | 0.794 | 0.013 | 0.352 |
| rostralmiddlefrontal thickness | -0.005 | 0.756 | 0.003 | 0.794 |
| postcentral thickness | 0.000 | 0.978 | 0.016 | 0.256 |
| lingual thickness | 0.012 | 0.385 | 0.026 | **0.041** |
| lateraloccipital thickness | 0.011 | 0.414 | 0.021 | 0.096 |
| pericalcarine thickness | 0.018 | 0.176 | 0.036 | **0.002** |
| cuneus thickness | 0.029 | **0.026** | 0.041 | **0.001** |
| **Subcortical area** |  |  |  |  |
| cerebellum Cortex | -0.032 | **0.010** | -0.029 | **0.020** |
| ventralDC | -0.026 | **0.027** | -0.028 | **0.020** |
| pallidum | -0.009 | 0.429 | -0.009 | 0.429 |
| vessel | -0.013 | 0.311 | -0.014 | 0.311 |
| thalamus proper | -0.009 | 0.442 | -0.013 | 0.311 |
| choroid plexus | -0.008 | 0.489 | -0.006 | 0.581 |
| hippocampus | -0.007 | 0.548 | -0.011 | 0.407 |
| accumbens area | 0.012 | 0.338 | 0.012 | 0.312 |
| lateral ventricle | 0.008 | 0.445 | 0.013 | 0.311 |
| amygdala | 0.009 | 0.442 | 0.016 | 0.211 |
| inferior lateral ventricle | 0.019 | 0.103 | 0.025 | **0.031** |
| putamen | 0.013 | 0.311 | 0.014 | 0.311 |
| caudate | 0.024 | **0.038** | 0.026 | **0.027** |
| brain stem | -0.026 | **0.027** |  |  |
| CC Posterior | -0.019 | 0.103 |  |  |
| CC Anterior | -0.013 | 0.311 |  |  |
| CC Mid Posterior | -0.003 | 0.706 |  |  |
| CC Central | -0.004 | 0.681 |  |  |
| fifth ventricle | -0.010 | 0.429 |  |  |
| CC Mid Anterior | 0.005 | 0.615 |  |  |
| forth ventricle | -0.006 | 0.576 |  |  |
| optic chiasm | -0.010 | 0.420 |  |  |
| third ventricle | 0.009 | 0.442 |  |  |

Model 1 were adjusted by age, sex, Townsend deprivation index, ethnicity, and wear-season. Model 2 were additionally adjusted by educational attainment, smoking status, alcohol consumption status, physical activity, and BMI.

*p* values were corrected for multiple comparisons using the false discovery rate (FDR).

# Table S6. Linear correlation coefficients of white matter tract-specific FA with relative amplitude.

| **Model 1** | **left** |  | **right** |  |
| --- | --- | --- | --- | --- |
| **White matter tract** | **coefficient** | ***P-value*** | **coefficient** | ***P-value*** |
| Acoustic radiation | -0.007 | 0.520 | 0.019 | 0.098 |
| Anterior thalamic radiation | -0.021 | 0.075 | -0.022 | 0.071 |
| Cingulate gyrus part of cingulum | -0.021 | 0.077 | -0.020 | 0.078 |
| Parahippocampal part of cingulum | -0.010 | 0.374 | -0.003 | 0.722 |
| Corticospinal tract | 0.009 | 0.397 | 0.017 | 0.143 |
| Inferior fronto-occipital fasciculus | -0.021 | 0.075 | -0.026 | **0.031** |
| Inferior longitudinal fasciculus | -0.036 | **0.002** | -0.028 | **0.021** |
| Medial lemniscus | 0.009 | 0.397 | -0.012 | 0.343 |
| Posterior thalamic radiation | -0.068 | **0.000** | -0.061 | **0.000** |
| Superior longitudinal fasciculus | -0.032 | **0.006** | -0.019 | 0.096 |
| Superior thalamic radiation | -0.016 | 0.150 | -0.008 | 0.466 |
| Uncinate fasciculus | -0.005 | 0.657 | -0.012 | 0.311 |
| Forceps major | -0.011 | 0.359 |  |  |
| Forceps minor | -0.023 | 0.066 |  |  |
| Middle cerebellar peduncle | 0.011 | 0.349 |  |  |

| **Model 2** | **left** |  | **right** |  |
| --- | --- | --- | --- | --- |
| **White matter tract** | **coefficient** | ***P-value*** | **coefficient** | ***P-value*** |
| Acoustic radiation | -0.015 | 0.195 | 0.000 | 0.998 |
| Anterior thalamic radiation | -0.019 | 0.102 | -0.023 | **0.042** |
| Cingulate gyrus part of cingulum | -0.022 | **0.046** | -0.017 | 0.142 |
| Parahippocampal part of cingulum | -0.003 | 0.930 | -0.001 | 0.983 |
| Corticospinal tract | 0.001 | 0.969 | -0.001 | 0.969 |
| Inferior fronto-occipital fasciculus | -0.023 | **0.042** | -0.032 | **0.004** |
| Inferior longitudinal fasciculus | -0.035 | **0.002** | -0.035 | **0.002** |
| Medial lemniscus | 0.004 | 0.831 | -0.012 | 0.296 |
| Posterior thalamic radiation | -0.049 | **0.000** | -0.054 | **0.000** |
| Superior longitudinal fasciculus | -0.031 | **0.006** | -0.025 | **0.034** |
| Superior thalamic radiation | -0.023 | **0.042** | -0.014 | 0.216 |
| Uncinate fasciculus | -0.010 | 0.401 | -0.015 | 0.216 |
| Forceps major | -0.012 | 0.296 |  |  |
| Forceps minor | -0.020 | 0.080 |  |  |
| Middle cerebellar peduncle | -0.003 | 0.930 |  |  |

Model 1 were adjusted by age, sex, Townsend deprivation index, ethnicity, and wear-season. Model 2 were additionally adjusted by educational attainment, smoking status, alcohol consumption status, physical activity, and BMI.

*p* values were corrected for multiple comparisons using the false discovery rate (FDR).
